# A novel vesivirus (family *Caliciviridae*) in European badgers (*Meles meles*) in Hungary, 2020/2021

**DOI:** 10.1007/s00705-023-05733-6

**Published:** 2023-03-11

**Authors:** Gábor Reuter, Péter Pankovics, Gábor Nagy, Sándor Szekeres, Ákos Boros

**Affiliations:** 1 Department of Medical Microbiology and Immunology Medical School, University of Pécs, Szigeti út 12, H-7624 Pécs, Hungary; 2 Department of Animal Physiology and Health, Hungarian University of Agriculture and Life Science, Kaposvár Campus, Kaposvár, Hungary; 3 Department of Parasitology and Zoology, University of Veterinary Medicine, Budapest, Hungary

## Abstract

In this study, a novel vesivirus (family *Caliciviridae*) was detected and characterized in faecal and tissue (blood and spleen) specimens collected from three (23.1%) out of 13 European badgers (*Meles meles*) in Hungary that were tested using RT-PCR and sequencing methods. The complete genome of the vesivirus strain European badger/B40/2021/HUN (OQ161773) is 8,375 nucleotides in length. The ORF1, ORF2, and ORF3 proteins have 81.1%, 70.5%, and 64.2% amino acid sequence identity, respectively, to the corresponding proteins of Asian badger vesivirus, which was first reported in badgers in China in 2022. These results indicate that more than one lineage/species of vesiviruses circulates in mustelid badgers in geographically different regions.

**Supplementary Information:**

The online version contains supplementary material available at 10.1007/s00705-023-05733-6.

The family *Caliciviridae* includes viruses with a linear, positive-sense single-stranded RNA genome of 6.4–8.5 kb with two to three open reading frames (ORFs). The family consists of 11 genera that include caliciviruses of mammals (*Lagovirus*, *Norovirus*, *Nebovirus*, *Recovirus*, *Sapovirus*, *Valovirus*, and *Vesivirus*), birds (*Bavovirus* and *Nacovirus*), and fish (*Minovirus* and *Salovirus*) [1]. Members of the genus *Vesivirus* have three ORFs. ORF1 encodes the nonstructural polyprotein (NS), which is post-translationally cleaved into six proteins. ORF2 and ORF3 encode the viral capsid proteins: the major capsid protein, VP1, and the minor basic protein, VP2, respectively. ORF1 and ORF2 are separated by either 2 nt (GC for feline calicivirus) or 5 nt (CCACT/C for marine vesiviruses) [1].

Vesiviruses have been identified in a wide range of terrestrial (e.g., cat, dog, swine, mink, rabbit, and cattle) and marine (e.g., San Miguel sea lion) mammals. However, there are only limited data available about vesivirus infections and diseases in humans [2–5]. Vesicular exanthema of swine virus (VESV) is the prototype vesivirus causing clinical signs of vesicles and fetal damage in swine. At present, there are two official vesivirus species, *Vesicular exanthema of swine virus* and *Feline calicivirus*, but several others are awaiting recognition [1]. Vesivirus has been reported recently in Asian badgers (*Meles leucurus*), which are frequently traded and consumed game animals in China [6].

Fifteen species of mustelid badgers are known. The European badger (*Meles meles*) is native to almost all of Europe, extending to the Volga River in the East. It is a carnivorous and highly adaptable and opportunistic omnivore whose diet encompasses a wide range of animals and plants. The European badger is found in deciduous and mixed woodlands, clearings, spinneys, pastureland, and scrub. It has also adapted to life in suburban areas and urban parks [7; https://en.wikipedia.org/wiki/European_badger].

In this study, a novel vesivirus was identified in faecal and tissue specimens collected from European badgers, and its complete genome sequence was determined.

A total of 13 faecal specimens were collected from European badgers (eight females and five males) between May 2020 and April 2021 in South Transdanubia (Baranya and Somogy Counties) in Hungary (Supplementary Table S1). Available tissue specimens (blood, thigh muscle, diaphragm, and spleen) from selected animals were also tested. Badgers were either legally hunted by a professional hunter with the approval of the national control program for wildlife or were found dead due collisions with vehicles.

Viral RNA was isolated using TRIzol Reagent (Thermo Fisher Scientific, Waltham, MA, USA) according to the manufacturer’s instructions. A conventional RT-PCR method was applied using a newly designed common vesivirus screening primer pair (vesivirus-screen-F, 5’-GCTGAAGCCGAGGGAAAGGTT-3’, corresponding to nt 3674–3694 of the study strain European badger/B40/2021/HUN, OQ161773, and vesivirus-screen-R, 5’-GCCTTYTGTCCCAACACATAG-3,’ corresponding to nt 3831 − 3811 of the study strain) based on the border of VPg/Pro of the genome sequences of mink calicivirus (MF677852), San Miguel sea lion calicivirus (KM244552), Asian badger vesivirus (OM480529), and ferret-badger vesivirus (KJ701554). The complete vesivirus genome was amplified and sequenced by various RT-PCR techniques (3’/5’ RACE PCR and long-range PCR) using primers designed based on the sequences of the closest vesivirus relatives and by the “primer-walking” method. PCR products were sequenced directly by the Sanger method using a BigDye Terminator v1.1 Cycle Sequencing Kit (Thermo Fisher Scientific), using an automated sequencer (AB3500 Genetic Analyzer, Applied Biosystems, Hitachi, Tokyo, Japan).

Three (specimens B10, B40, and B41) of the 13 faecal samples from European badger were RT-PCR-positive for vesivirus (Supplementary Table S1). The complete genome sequence of European badger vesivirus strain European badger/B40/2021/HUN (OQ161773) was determined from one selected sample (B40). The European badger/B40/2021/HUN genome is 8,375 nt long, excluding the poly(A) tail (Fig. 1). The genome has three ORFs. ORF1 is 5,742 nt long and encodes a 1,913-aa-long nonstructural NS polyprotein with 81.1% aa sequence identity (query coverage, 99%) to the corresponding nonstructural polyprotein of Asian badger vesivirus (OM480529), its closest match in the GenBank database. ORF2 is 2,106 nt long and it encodes a 701-aa-long VP1 capsid protein with 70.5% aa sequence identity (query coverage, 100%) to the corresponding VP1 protein of Asian badger vesivirus (OM451121), its closest match in the GenBank database. Alignment of the aa sequence revealed the presence of a potential leader capsid (LC) of VP1 with the capsid cleavage site FRAE_170_/S (Fig. 1). ORF3 is 411 nt long and encodes a 136-aa-long VP2 minor basic capsid protein with 64.2% aa sequence identity (query coverage, 90%) to the corresponding VP2 protein of Asian badger vesivirus (OM480529), its closest match in the GenBank database. ORF1 and ORF2 of European badger vesivirus are separated by 3 nt (GCG instead of GC for Asian badger vesivirus), such that ORF1 and ORF2 are in the same reading frame (Fig. 1). The 5’ and 3’ untranslated regions (UTR) are 13 and 104 nt long, respectively.

**Fig. 1:**
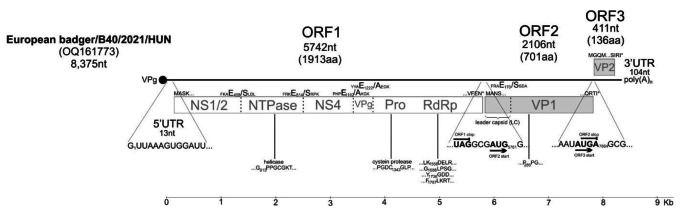
Schematic genome organization (line) and protein products (box) of European badger vesivirus strain European badger/B40/2021/HUN (OQ161773). The complete genome and protein map are drawn to scale. Nucleotide (nt) sequences of the extreme 5’ untranslated region (UTR) and the border of the open reading frames ORF1/ORF2 and ORF2/ORF3 are shown with stop and start codons. Also shown are the conserved caliciviral amino acid (aa) motifs, first and last amino acids of the ORFs, and the predicted P4/P4’ protein cleavage sites

The three European badger vesivirus isolates from this study (B10, B40, and B41) differed in their nucleotide sequences. In the 116-nucleotide-long amplified common region (ORF1), there was 89–93% nucleotide sequence identity between the strains. The 4,703-long 3’ part (partial ORF1, complete ORF2/ORF3 and 3’UTR) of the European badger/B10/2020/HUN (OQ161774) and European badger/B41/2021/HUN (OQ161775) genomes were also sequenced. The ORF2 of European badger/B10/2020/HUN and European badger/B41/2021/HUN (OQ161775) showed 84/85% nt and 94/95% aa sequence identity, respectively to the corresponding nt and aa sequences of the strain European badger/B40/2021/HUN.

Phylogenetic analysis based on amino acid sequences of the NS (ORF1) and VP1 (ORF2) proteins showed that the European badger vesiviruses were clustered together with vesiviruses from Asian badgers and ferret badgers (*Melogale moschata*) from China, with the three European badger vesivirus variants forming a distinct lineage (Fig. 2).

**Fig. 2:**
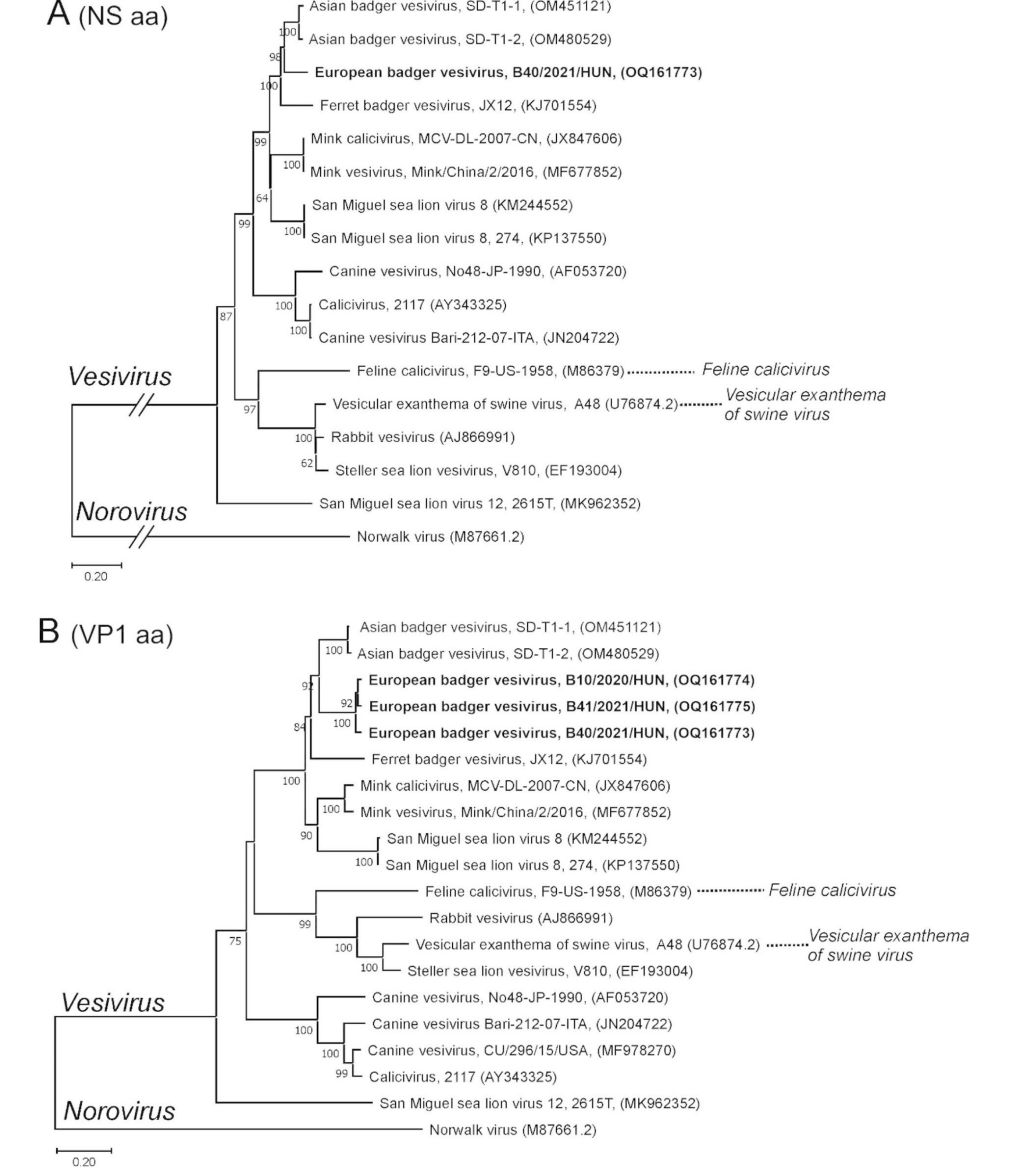
Phylogenetic analysis based on amino acid sequences of (A) NS (ORF1) and (B) VP1 (ORF2) proteins of the study strains European badger/B40/2021/HUN (OQ161773), European badger/B10/2020/HUN (OQ161774), and European badger/B41/2021/HUN (OQ161775) (in bold) and representative members of the genus *Vesivirus*. Norwalk virus (genus *Norovirus*) was used as an outgroup. Neighbor-joining trees were generated from MUSCLE-based amino acid (aa) alignments using the Jukes-Cantor model and 1000 bootstrap replicates in MEGA 11 software [14]. The scale bars indicate the number of aa substitutions per site. The designation of the sequences is as follows: virus name, isolate or strain name, accession number in brackets.The two officially classified vesivirus species are indicated by dotted lines

Available tissue samples (blood, thigh muscle, diaphragm, and spleen) from animals (B10, B40, and B41) that had vesivirus-positive faecal specimens were also investigated by RT-PCR using the screening primers. Vesivirus RNA was found in all of the blood (N = 3) and spleen (N = 3) samples by RT-PCR and sequencing; however, viral RNA was not detected in muscle (thigh muscle and diaphragm) specimens (Supplementary Table S1).

Vesiviruses have been shown to be capable of infecting a variety of terrestrial and marine mammal host species, raising concern about their zoonotic potential [2–5]. In this study, a novel vesivirus from European badgers in Europe was identified and characterized.

The European badger vesivirus forms a common (phylo)genetic cluster with vesiviruses from Asian badger [6], Chinese ferret-badger [8], mink [9], and San Miguel sea lion (SMSV8, [10]; Knowles et al., unpublished). While the European badger vesivirus genome sequence had the highest sequence similarity to a vesivirus identified recently in Asian badgers in China, these two groups of badger vesiviruses are significantly different genetically (and likely antigenically), with 29.5% aa sequence difference in VP1. This indicates that more than one lineage or species of vesiviruses circulates in mustelid badgers in geographically different regions.

There are only two officially recognized vesivirus species in the genus *Vesivirus* (family *Caliciviridae*): the species *Vesicular exanthema of swine virus* and the species *Feline calicivirus* (https://talk.ictvonline.org/ictv-reports/ictv_online_report/positive-sense-rna-viruses/w/caliciviridae). However, several further unclassified vesiviruses from different host species are awaiting species classification, including a cluster of vesiviruses from dogs, minks, San Miguel sea lions (SMSV types 8 and 12), badgers, and ferrets.

Some types of vesiviruses have been associated with skin lesions, hepatitis, abortions, pneumonia, diarrhoea, and encephalitis in animals [11–13] and humans [2]. Skin and mucosal diseases are associated with itching vesicles, blisters, or skin ulcers [2, 13]. In our study, vesivirus RNA was detected not only in faecal specimens but also in available tissue samples (blood and spleen) in all three vesivirus-infected badgers. This suggests that the badger is a host species of this vesivirus and that viral RNA (or infectious virions) enters into the bloodstream and may cause viremia and generalized infections. In our study, two of the three animals infected with vesivirus were clinically healthy without visible skin lesions (blisters or ulcers); however, animal B40 had a minor skin abrasion of unknown origin/aetiology (potentially from physical injuries) in the neck area.

In contrast to other caliciviruses (e.g., noroviruses), some types of vesiviruses receive relatively little clinical and scientific attention. A common screening RT-PCR primer pair (Vesivirus-screen-F/R) was designed in this study based on the cluster of known vesivirus genome sequences from badgers, ferret-badgers, and minks and the SMSV8 sequence from San Miguel sea lions, which are suitable for molecular epidemiological studies and further discovery of novel vesiviruses.

## Electronic supplementary material

Supplementary Table S1
