# Supplementary material for: A novel vesivirus (family Caliciviridae) in European badgers (Meles meles) in Hungary, 2020/2021
Source: Arch Virol. 2023 Mar 11;168(4):108. doi: 10.1007/s00705-023-05733-6 (PMC10006033; doi:10.1007/s00705-023-05733-6)
Supplement: Supplementary file 1 — Supplementary Table S1 [file 705_2023_5733_MOESM1_ESM.doc]

**Table S1.** Detailed background information of European badgers and faecal samples used for the epidemiological investigations of vesivirus as well as the results of vesivirus RT-PCR screening. ID: identification marks. Vesivirus sequences were deposited in the GenBank database. n.a.= not available

| **Sample ID** | **Sample collection date (d/m/y)** | **Geolocation of the sample collection (lat./long.)** | **County** | **Sex** | **Age (years)** | **Health status** | **RT-PCR for vesivirus, faeces (GenBank Acc. No)** | **RT-PCR for vesivirus, tissue samples** |
| --- | --- | --- | --- | --- | --- | --- | --- | --- |
| **B1** | 07.05.2020. | 46.226621/  17.727019 | Baranya | male | >1 | no obvious health problem; condition: moderate | negative | n.a. |
| **B2** | 22.05.2020. | 46.195223/  17.774439 | Baranya | female | >1 | no obvious health problem; condition: good | negative | n.a. |
| **B3** | 01.06.2020. | 46.240844/  17.760548 | Somogy | female | >1 | no obvious health problem; condition: good | negative | n.a. |
| **B4** | 07.06.2020. | 46.295382/  17.729825 | Somogy | male | <1 | no obvious health problem; condition: good | negative | n.a. |
| **B5** | 09.06.2020 | 46.200178/  17.805042 | Baranya | female | >1 | no obvious health problem; condition: good | negative | n.a. |
| **B6** | 18.06.2020. | 46.223511/  17.780256 | Somogy | male | >1 | no obvious health problem; condition: moderate | negative | n.a. |
| **B7** | 08.07.2020. | 46.270491/  17.756975 | Somogy | male | >1 | skin abrasion in the neck and on the front legs; condition: good | negative | n.a. |
| **B9** | 17.07.2020. | 45.8061/  17.8567 | Baranya | female | >1 | no obvious health problem; condition: good | negative | n.a. |
| **B10** | 17.07.2020. | 45.8185/  17.8536 | Baranya | female | >1 | no obvious health problem; condition: good | **positive** (OQ161774) | **blood: positive**; thigh muscle: negative; diaphragm: negative; **spleen: positive** |
| **B12** | 17.07.2020. | 45.8323/  17.8458 | Baranya | female | >1 | no obvious health problem; condition: good | negative | n.a. |
| **B13** | 22.07.2020. | 46.254181/  17.745881 | Somogy | female | >1 | no obvious health problem; condition: good | negative | n.a. |
| **B40** | 21.04.2021. | 46.308668/  17.772815 | Somogy | male | >1 | skin abrasion in the neck (potentially from physical injuries); condition: good | **positive** (OQ161773) | **blood: positive**; thigh muscle: negative; diaphragm: negative; **spleen: positive** |
| **B41** | 24.04.2021. | 46.237702/  17.760236 | Somogy | female | >1 | no obvious health problem; condition: good | **positive** (OQ161775) | **blood: positive**; thigh muscle: negative; diaphragm: negative; **spleen: positive** |
